# Drug-eluting beads TACE is safe and non-inferior to conventional TACE in HCC patients with TIPS

**DOI:** 10.1007/s00330-021-07834-9

**Published:** 2021-04-24

**Authors:** Wenzhe Fan, Jian Guo, Bowen Zhu, Shutong Wang, Lei Yu, Wanchang Huang, Huishuang Fan, Fuliang Li, Yanqin Wu, Yue Zhao, Yu Wang, Miao Xue, Hongyu Wang, Jiaping Li

**Affiliations:** 1 Department of Interventional Oncology, The First Affiliated Hospital of Sun Yat-Sen University, 58 Zhongshan 2nd Road, Guangzhou, 510080 People’s Republic of China; 2 Department of Hepatic Surgery, The First Affiliated Hospital of Sun Yat-Sen University, Guangzhou, People’s Republic of China; 3 Department of Interventional Radiology, The People’s Hospital of Guangxi Zhuang Autonomous Region, Guangxi, People’s Republic of China; 4 Department of Interventional Radiology, The First People’s Hospital of Yulin, Guangxi, People’s Republic of China; 5 Interventional Department, Dongguan People’s Hospital, Dongguan, People’s Republic of China; 6 Liver and Gall Surgical Department, Gaozhou People’s Hospital, Gaozhou, People’s Republic of China

**Keywords:** Chemmembolizatioin, therapeutic, Carcinoma, hepatocellular, Portasystemic shunt, transjugular intrahepatic

## Abstract

**Objectives:**

This study aims to compare the safety and effectiveness between transarterial chemoembolization (TACE) with drug-eluting beads (DEB-TACE) and conventional TACE (cTACE) using lipiodol-based regimens in HCC patients with a transjugular intrahepatic portosystemic shunt (TIPS).

**Methods:**

This retrospective study included patients with patent TIPS who underwent TACE from January 2013 to January 2019 that received either DEB-TACE (DEB-TACE group, *n* = 57) or cTACE (cTACE group, *n* = 62). The complications, liver toxicity, overall survival (OS), time to progression (TTP), and objective response rate (ORR) were compared between the groups.

**Results:**

Altogether, 119 patients (50 ± 11 years, 107 men) were evaluated. The incidence of adverse events, including abdominal pain within 7 days (45.6% vs 79.0%, *p* < 0.001) and hepatic failure within 30 days (5.3% vs 19.4%, *p* = 0.027), were significantly lower in the DEB-TACE group than in the cTACE group. Compared to the cTACE group, the DEB-TACE group also showed mild liver toxicities in terms of increased total bilirubin (8.8% vs 22.6%), alanine aminotransferase (5.3% vs 21.0%), and aspartate aminotransferase (10.5% vs 29.0%) levels. The DEB-TACE group had better ORR than the cTACE group (70.2% vs 50.0%). The median OS and TTP were longer in the DEB-TACE group (11.4 vs 9.1 months, hazard ratio [HR] = 2.46, *p* < 0.001; 6.9 vs 5.2 months, HR = 1.47, *p* = 0.045). Multivariable analysis showed that α-fetoprotein levels, Barcelona clinic liver cancer stage, and treatment allocation were independent predictors of OS.

**Conclusion:**

DEB-TACE is safe and effective in HCC patients with a TIPS and is potentially superior to cTACE in terms of complications, liver toxicities, OS, TTP, and ORR.

**Key Points:**

• *DEB-TACE is safe and effective in HCC patients after a TIPS procedure.*

• *DEB-TACE improves overall survival, objective response rate, and liver toxicities and is non-inferior to cTACE in terms of time to progression.*

• *DEB-TACE might be a potential new therapeutic option for HCC patients with TIPS.*

**Supplementary Information:**

The online version contains supplementary material available at 10.1007/s00330-021-07834-9.

## Introduction

Hepatocellular carcinoma (HCC) is the most frequent primary liver malignancy and the third leading cause of cancer-related mortality [1]. Transarterial chemoembolization (TACE) is an established treatment for unresectable HCC [2]. However, chemoembolization might lead to hepatic dysfunction and increase liver toxicity, which restricts the usage of TACE in some HCC patients [3]. Besides being a risk factor for the development of HCC, liver cirrhosis predisposes patients to portal hypertension [4]. Transjugular intrahepatic portosystemic shunt (TIPS) is an important treatment strategy in managing portal hypertension complications, including variceal bleeding and refractory ascites [5]. Some HCC patients with portal hypertension treated with TIPS still require treatment for liver malignancy. However, because of the diversion of the portal venous flow via the TIPS, TACE is not regarded as the first therapeutic choice for such patients. Theoretically, conventional TACE (cTACE) can further reduce liver perfusion, which might lead to the increased liver deterioration [6, 7]. Although repeated cTACE can be safely performed in selected patients with TIPS, the rate of grade 3 or 4 severe adverse events (SAEs) within 1 month is high (36.0%) [8]. According to previous research, the efficacy profile of cTACE in TIPS patients depended on the postprocedural complications [8].

Drug-eluting beads TACE (DEB-TACE), a variant of cTACE, selectively delivers a large amount of chemotherapeutic agents to the target over an extended period of time, minimizing the blood concentration of the drugs and related systemic effects, and reducing the embolic agents, which makes DEB-TACE more likely to have a positive influence in protecting the blood perfusing from the hepatic artery to the normal liver tissue [9]. Although a recent systematic review revealed that DEB-TACE fails to increase the survival advantage over cTACE [10], it was noted in the PRECISION V trial that drug-eluting beads loaded with doxorubicin (DEBDOX) showed lower incidences of systemic adverse events (AEs) and hepatotoxicity than cTACE [11]. The main difference in DEB-TACE and cTACE is that embolic material like DC beads in DEB-TACE remains within the arteries, whereas lipiodol used during cTACE may crosses the sinusoids into the portal venules, which may cause ischemia [12], and the dual embolic hit may be too much in the context of TIPS.

Presently, studies of TACE in HCC patients who underwent functional TIPS procedures are limited [13–15], and only cTACE was used in previous reports. Thus, our research aimed to compare the adverse effects, local response, and long-term survival between patients with TIPS receiving DEB-TACE and those receiving cTACE. The hypothesis is that, with the lower incidences of systemic adverse events and hepatotoxicity, DEB-TACE might be more effective and safer for HCC patients with TIPS compared with cTACE

## Materials and methods

### Study design

This retrospective study collected data between January 2013 to January 2019 from five tertiary medical centers (The First Affiliated Hospital, Sun Yat-sen University, Guangzhou, China; The People’s Hospital of Guangxi Zhuang Autonomous Region, Guangxi, China; The First People’s Hospital of Yulin, Guangxi, China; Dongguan People’s Hospital, Dongguan, China; and Gaozhou People’s Hospital, Gaozhou, China). Approval was obtained from the relevant Institutional Review Board. The requirement for informed consent from the patients was waived due to the retrospective nature of this study. The main objectives were to evaluate complications, liver toxicities, overall survival (OS), time to progression (TTP), disease control rate (DCR), and objective response rate (ORR) of DEB-TACE.

### Patients

The eligibility criteria were as follows: (*a*) age of 18–75 years; (*b*) HCC diagnosed before TIPS according to the American Association for Liver Disease and European/American Association for Liver Disease guidelines [16, 17]; (*c*) patients who underwent a TIPS procedure as the secondary prevention of variceal bleeding or refractory ascites; (*d*) patients who had their first TACE procedure performed at our institutions and had a patent portal vein vascular perfusion that was exhibited throughout the stent with mid stent Doppler velocity of > 60 cm/s within 1 month after TIPS procedure [18]; (*e*) Eastern Cooperative Oncology Group (ECOG) performance status score of 0 or 1; and (*f*) Child-Pugh A-B class. The exclusion criteria were as follows: (*a*) portal vein tumor thrombus (PVTT) in the main portal vein; (*b*) liver transplant after TIPS or the treatment for the malignancy including resection or ablation; (*c*) severe dysfunction of the heart, kidney, or other organs; and (*d*) contraindication for TACE because of severe coagulation disorders or hepatic encephalopathy.

### TACE procedures

All procedures were performed by experienced interventional radiologists. Standard angiographic facilities and protocols were used for hepatic angiography and catheterization. Imaging of the celia and superior mesenteric arteries was performed in all patients to evaluate the liver vasculature circulation prior to treatment. Super-selective catheterizations were performed in every embolization of DEB-TACE or cTACE procedure if possible. However, in patients with bilobar multinodular disease, lobar artery was selective to catheterize at least.

For cTACE, a solution containing a mixture of 50-mg doxorubicin (Adriamycin; Pharmacia & Upjohn) with Lipiodol (Guerbet) was infused, followed by the infusion of 300–500-μm trisacryl gelatin microspheres (Embosphere particles; Biosphere Medical) until stasis was nearly achieved.

For DEB-TACE, DEB usage was as recommended [19]. The DC Bead™ particles (Biocompatibles) used in the present study were 100–300 or 300–500 μm in size. Each vial of DC Bead™ (2 mL of beads) was loaded with 75-mg doxorubicin dissolved in sterilized water. After loading for 30 min, at least 5–10 mL of nonionic isotonic contrast (270-mg/mL Visipaque [iodixanol]) was injected into the vial per 1 mL of DEBDOX. The 10-mL suspension of DEBDOX was then aspirated into a syringe and injected in a consistent manner [19]. The embolization protocols performed in our research only rarely necessitated supplementary embolic materials to avoid DEBDOX overdose, and 300–500-μm Embosphere microspheres were required for complete devascularization.

DEBDOX doses were adjusted according to the tumor diameter (based on the ellipsoid volume, i.e., height × width × length × π/6). The endpoint of primary chemoembolization was the complete devascularization of the HCC observed on angiograms [19, 20].

### Assessment of outcomes and safety

OS time was measured from first cTACE or DEB-TACE after TIPS to death or last follow-up. TTP was defined as the time from the day of first cTACE or DEB-TACE until the detection of progressive disease (PD). Patients were followed up once a month. Within 1 week prior to the treatment, all patients underwent triphasic contrast-enhanced computed tomography (CT) or magnetic resonance imaging (MRI), and all parameters including serum α-fetoprotein (AFP) level and hepatic function were documented. Tumor response and safety were assessed at 1-month intervals, until death or the complete refractory to cTACE and DEB-TACE. Once any residual tumor or recurrence was observed, an additional cTACE or DEB-TACE would be performed according to the criteria described above. The modified Response Evaluation Criteria in Solid Tumors (mRECIST) were used to assess the efficacy of local tumor response according to images acquired 1 month after TACE [21]. In each institution, measurements were performed by two independent radiologists from the Department of Medical Imaging, with consensus review by a third experienced radiologist performed for equivocal cases. Triphasic contrast-enhanced CT and MRI were used for imaging follow-up. The best overall response during treatment was considered the final response. The last follow-up date was September 30, 2019.

AEs were classified according to the adverse event classification proposed by the Society of Interventional Radiology (SIR) standards of practice committee [22]. SAEs were defined as severe AE or life-threatening or disabling event, namely AE severity of grade 3 or 4 in SIR classification, within 1 month after TACE. Liver toxicity was evaluated using Common Terminology Criteria of Adverse Events v4.0 [23]. Hepatic reserve function was evaluated with the albumin-bilirubin (ALBI) score. The ALBI score was calculated on the basis of the total bilirubin (TBil) and serum albumin (ALB) levels using the following formula: ALBI score = (− 0.085 × ALB [g/L]) + (0.66 × log10 TBil [μmol/L]), and the ALBI score was categorized into three grades based on the following scores: ≤ −2.60 = grade 1, > −2.60 to ≤ −1.39 = grade 2, and > −1.39 = grade 3 [24].

### Statistical analysis

Comparisons between two groups were assessed by using Student’s *t* test for continuous variables, expressed as the mean ± standard deviation, and Pearson’s chi-squared (*χ*^2^) test for categorical data, presented as a frequency. Survival curves were assessed by Kaplan–Meier analyses, with univariable analysis performed using the log-rank test. Multivariable analysis was carried out by Cox regression analysis for variables that were significant at *p* < 0.05 on the univariable analysis. All statistical tests were two-sided, and a *p* value of < 0.05 was considered to demonstrate statistical significance.

## Results

### Baseline characteristics

**Fig. 1:**
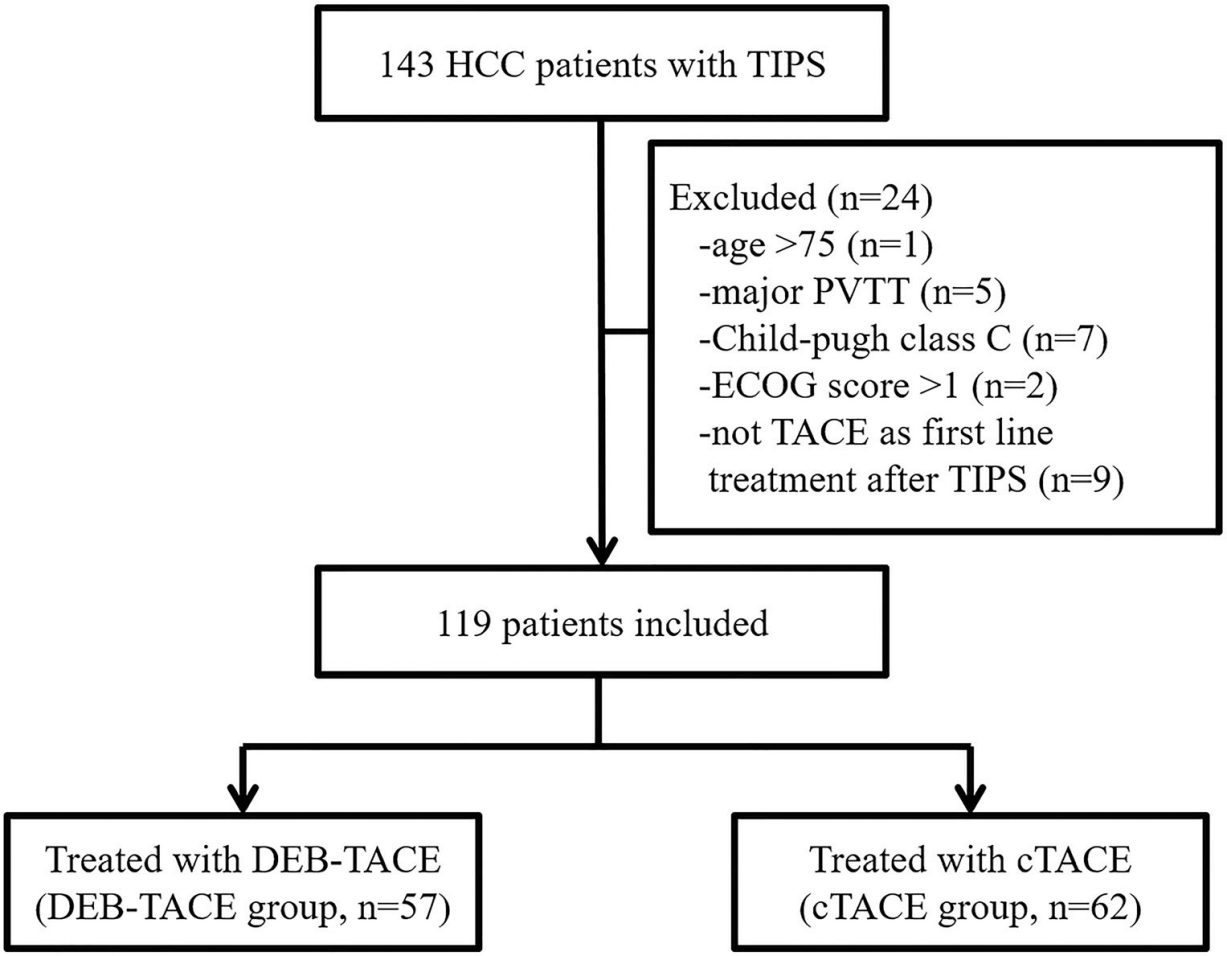
Flow chart showing the selection of patients. cTACE, conventional TACE; DEB-TACE, drug-eluting beads transarterial chemoembolization; ECOG, Eastern Cooperative Oncology Group; PVTT, portal vein tumor thrombus; TACE, transcatheter arterial chemoembolization; TIPS, transjugular intrahepatic portosystemic shunt

A retrospective review of the records of 143 consecutive HCC patients with TIPS between January 2013 and January 2019 was performed. Finally, 119 patients were enrolled. DEB-TACE was consented to by 57 patients (DEB-TACE group), whereas the remaining 62 patients consented to undergo cTACE (cTACE group) (Fig. 1). Table 1 shows the balanced baseline characteristics between the DEB-TACE and cTACE groups. The corresponding average lengths of hospital stay were 5.3 and 5.7 days respectively. The DEB-TACE group underwent a total of 129 TACE sessions, whereas the cTACE group underwent 134 administrations of TACE in total (*p* = 0.601). The median follow-up duration was 9.1 months (range, 3.1−36.5 months) and 11.4 months (range, 3.1−26 months) in the DEB-TACE and cTACE groups, respectively. The median size of the largest tumor was 7.3 cm (range, 1.3−15.8 cm). No significant differences were observed in the baseline characteristics between the two groups.

**Table 1:** Comparison of characteristics between patients in the DEB-TACE group and cTACE group

Characteristics	Number (%)/Median (IQR) ^*a*^			*p* value
	Total (*n* = 119)	DEB-TACE group (*n* = 57)	cTACE group (*n* = 62)	
Age (y)	50 ± 11	51 ± 12	49 ± 10	0.118
< 50	59 (49.6%)	24 (42.1%)	35 (56.5%)	
≥ 50	60 (50.4%)	33 (57.9%)	27 (43.5%)	
Sex				0.445
Male	107 (89.9%)	50 (87.7%)	57 (91.9%)	
Female	12 (10.1%)	7 (12.3%)	5 (8.1%)	
HBV				0.445
Absence	12 (10.1%)	7 (12.3%)	5 (8.1%)	
Presence	107 (89.9%)	50 (87.7%)	57 (91.9%)	
Previous radical treatment^*b*^				0.832
Absence	91 (76.5%)	43 (75.4%)	48 (77.4%)	
Presence	28 (23.5%)	14 (24.6%)	14 (22.6%)	
Length of hospital stay	6 (4-8)	6 (4-8)	6 (4-8)	0.906
TACE times				0.920
Once	35 (29.4%)	17 (29.8%)	18 (29.1%)	
Twice	38 (31.9%)	19 (33.4%)	19 (30.6%)	
More	46 (38.7%)	21 (36.8%)	25 (40.3%)	
ECOG score				0.486
0	85 (71.4%)	39 (68.4%)	46 (74.2%)	
1	34 (28.6%)	18 (31.6%)	16 (25.8%)	
TIPS indication				0.166
Variceal bleeding	96 (80.7)	43 (75.4)	53 (85.5)	
Refractory ascites	23 (19.3)	14 (24.6)	9 (14.5)	
WBC (×10^9^/L)	5.7 (4.8–7.4)	5.8 (4.8–7.4)	5.7 (4.5–7.0)	0.597
RBC (×10^12^/L)	4.3 (3.8–4.9)	4.6 (4.0–5.0)	4.2 (3.8–4.7)	0.449
Platelet count (×10^9^/L)	137 (99–206)	151 (100–212)	135 (97–162)	0.152
AFP (ng/ml)	155 (22–1441)	386 (11–1741)	112 (27–773)	0.237
< 200	61 (51.3%)	26 (45.6%)	35 (56.5%)	
≥ 200	58 (48.7%)	31 (54.4%)	27 (43.5%)	
ALT (IU/L)	34 (23–56)	33 (22–52)	35 (27–58)	0.865
AST (IU/L)	53 (32–87)	54 (33–87)	51 (30–87)	0.690
ALB (g/L)	36.0 (31.7–39.6)	36.6 (33.0–40.7)	35.5 (30.8–38.1)	0.096
TBil (μmol/L)	19.0 (13.7–28.5)	21.7 (13.2–29.6)	18.6 (14.0–26.6)	0.357
PT (s)	13.9 (13.0–15.2)	13.7 (12.6–15.6)	14.0 (13.2–15.0)	0.849
ALBI score	–1.13 [(–1.51)–(–0.68)]	–1.24 [(–1.57)–(–0.57)]	–1.08 [(–1.41)–(–0.69)]	0.363
ALBI grade				0.136
2	40 (33.6%)	23 (57.5%)	17 (42.5%)	
3	79 (66.4%)	34 (43.0%)	45 (57.0%)	
Child-Pugh class				0.619
A	84 (70.6%)	39 (68.4%)	45 (72.6%)	
B	35 (29.4%)	18 (31.6%)	17 (27.4%)	
Intrahepatic tumors number				0.330
≤ 3	17 (14.3%)	10 (17.5%)	7 (11.3%)	
> 3	102 (85.7%)	47 (82.5%)	55 (88.7%)	
Tumor size ^*c*^ (cm)	7.3 (4.1–10.3)	6.0 (3.9–9.3)	8.2 (4.7–10.7)	0.269
< 5 cm	32 (29.1%)	18 (29.9%)	14 (35.3%)	
≥ 5 cm	87 (70.9%)	39 (70.1%)	48 (64.7%)	
PVTT				0.191
Absence	47 (39.5%)	26 (45.6%)	21 (33.9%)	
Presence	72 (60.5%)	31 (54.4%)	41 (66.1%)	
Extrahepatic metastasis				0.110
Absence	95 (79.8%)	49 (86.0%)	46 (74.2%)	
Presence	24 (20.2%)	8 (14.0%)	16 (25.8%)	
BCLC stage				0.192
A	8 (6.7%)	4 (7.1%)	4 (6.5%)	
B	37 (31.1%)	21 (36.8%)	16 (25.8%)	
C	74 (62.2%)	32 (56.1%)	42 (67.7%)	

^*a*^Median with interquartile range is shown for quantitative variables, whereas counts with proportions are shown for categorical variables.^*b*^ Radical treatment, treatment included liver transplant, resection, and ablation

^*c*^Tumor size, size of the largest tumor

*AFP*, alpha-fetoprotein; *ALB*, albumin; *ALBI*, albumin-bilirubin; *ALT*, alanine aminotransferase; *AST*, aspartate aminotransferase; *BCLC*, Barcelona Clinic Liver Cancer; *cTACE*, conventional transarterial chemoembolization; *DEB-TACE*, drug-eluting beads transarterial chemoembolization; *ECOG*, Eastern Cooperative Oncology; *HBV*, hepatitis B virus; *PLT*, platelet count; *PT*, prothrombin time (international ratio); *PVTT*, portal vein tumor thrombus; *RBC*, red blood cells; *TACE*, transarterial chemoembolization; *TBil*, total bilirubin; *TIPS*, transjugular intrahepatic portosystemic shunt; *WBC*, white blood cells

The TIPS procedure was performed on 96 and 23 patients for the secondary prevention of variceal bleeding and refractory ascites, respectively, according to a standard technique described previously [25]. The GORE VIATORR stent graft (W.L. Gore & Associates, Flagstaff) was applied to create the shunt in 67 patients, whereas the WALLSTENT (Boston Scientific) was used in 52 patients to form the TIPS. Supplemental Figures 1 and 2 show the treatment and imaging follow-up of two representative cases.

### Adverse events and liver toxicity

AEs within 7 days and SAEs within 30 days, related to DEB-TACE and cTACE, are exhibited in Tables 2 and 3. No treatment-related mortality occurred within 30 days after the final procedure. Supplementary post hoc analysis demonstrated that the incidence of abdominal pain within 7 days of the procedure was lower in the DEB-TACE group than in the cTACE group (45.6% vs 79.0%, *p* < 0.001, respectively) (Table 2). Myelosuppression and hepatic failure were the only two SAEs observed. The incidence of grade 3 or 4 treatment-related complications and liver toxicities within 30 days of the procedure was consistently lower for patients with hepatic failure (5.3% vs 19.4%, *p* = 0.027). The increases in the TBil, alanine aminotransferase (ALT), and aspartate aminotransferase (AST) levels were lower in the DEB-TACE group than in the cTACE group (8.8% vs 22.6%, *p* = 0.047; 5.3% vs 21.0%, *p* = 0.015; 10.5% vs 29.0%, *p* = 0.021, respectively) (Table 3).

**Table 2:** Incidence of adverse events within 7 days of a procedure between patients in the DEB-TACE group and the cTACE group after TIPS

Outcome	Number (%)/median (IQR) ^*a*^			*p* value
	Total (*n* = 119)	DEB-TACE group (*n* = 57)	cTACE group (*n* = 62)	
Fever ^***b***^	71 (59.7%)	30 (52.6%)	41 (66.1%)	0.141
Grade 1	46 (38.7%)	22 (38.6%)	24 (38.7%)	
Grade 2	20 (16.8%)	71 (12.3%)	13 (21.0%)	
Grade 3	6 (3.4%)	0 (1.8%)	3 (4.8%)	
Grade 4	1 (0.8%)	0	1 (1.6%)	
Abdominal pain ^*c*^	75 (63.0%)	26 (45.6%)	49 (79.0%)	< 0.001
Grade 1	44 (37.0%)	19 (33.3%)	25 (40.3% )	
Grade 2	22 (18.5%)	5 (8.8%)	17 (27.4%)	
Grade 3	9 (7.6%)	2 (3.5%)	7 (11.3%)	
Vomiting ^***b***^	46 (38.7%)	21 (36.8%)	25 (40.3%)	0.711
Grade 1	28 (23.5%)	15 (26.3%)	13 (21.0%)	
Grade 2	15 (12.6%)	5 (8.8%)	10 (16.1%)	
Grade 3	3 (2.5%)	1 (1.8%)	2 (3.2%)	
Grade 4	0	0	0	
TBil (μmol/L)	42.4 (30.7–58.7)	35 (24.8–45.3)	49.7 (38.1–63.5)	0.008
ALT (IU/L)	88 (65–151)	66 (44–89)	122.5 (77–198)	0.001
AST (IU/L)	105 (67–169)	87 (65–144)	145 (85–212)	0.004

^*a*^Median with interquartile range are shown for quantitative variables, whereas counts with proportions are shown for categorical variables

^*b*^No grade 5 patients recorded

^*c*^Only grades 1 to 3 according to the Common Terminology Criteria of Adverse Events v4.0

*ALT*, alanine aminotransferase; *AST*, aspartate aminotransferase; *cTACE*, conventional transarterial chemoembolization; *DEB-TACE*, drug-eluting beads transarterial chemoembolization; *TBil*, total bilirubin

**Table 3:** Incidence of serious adverse events within 30 days of a procedure between patients in the DEB-TACE group and the cTACE group after TIPS

Outcome (number of patients)	Total (*n* = 119)	DEB-TACE group (*n* = 57)	cTACE group (*n* = 62)	*p* value
Myelosuppression	13 (10.9%)	8 (8.8%)	12 (12.9%)	0.563
Grade 3	11 (9.2%)	7 (7.0%)	10 (11.3%)	
Grade 4	2 (1.7%)	1 (1.8%)	1 (1.6%)	
Hepatic failure	15 (12.6%)	3 (5.3%)	10 (19.4%)	0.027
Grade 3	13 (10.9%)	3 (5.3%)	8 (16.1%)	
Grade 4	2 (1.7%)	0	2 (3.2%)	
TBil increase	19 (16.0%)	5 (8.8%)	19 (22.6%)	0.047
Grade 3	17 (14.3%)	5 (8.8%)	17 (19.4%)	
Grade 4	2 (1.7%)	0	2 (3.2%)	
ALT increase	16 (13.4%)	3 (5.3%)	13 (21.0%)	0.015
Grade 3	16 (13.4%)	3 (5.3%)	13 (21.0%)	
Grade 4	0	0	0	
AST increase	24 (20.2%)	6 (10.5%)	18 (29.0%)	0.021
Grade 3	23 (19.3%)	6 (10.5%)	17 (27.4%)	
Grade 4	1 (0.8%)	0	1 (1.6%)	

*ALT*, alanine aminotransferase; *AST*, aspartate aminotransferase; *cTACE*, conventional transarterial chemoembolization; *DEB-TACE*, drug-eluting beads transarterial chemoembolization; *TBil*, total bilirubin

**Fig. 2:**
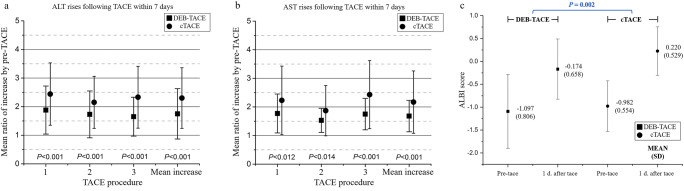
The comparison of changes in (**a**) ALT and (**b**) AST within 1 week after TACE and (**c**) ALBI score in the DEB-TACE and cTACE groups. ALT, alanine aminotransferase; AST, aspartate aminotransferase; cTACE, conventional transarterial chemoembolization; DEB-TACE, drug-eluting beads transarterial chemoembolization

For patients who underwent more than one TACE session, postoperative increases in the ALT and AST levels within 7 days of the procedure were considerably lower in the DEB-TACE group than in the cTACE group. The mean increase ratios of ALT and AST at baseline before chemoembolization were lower in the DEB-TACE group than in the cTACE group (1.75 vs 2.30, *p* < 0.001; 1.68 vs 2.17, *p* < 0.001) (Fig. 2a and b). The mean ALBI score increase after chemoembolization was lower in the DEB-TACE group than in the cTACE group (0.923 vs 1.202, *p* = 0.002) (Fig. 2c).

### Initial efficacy

**Fig. 3:**
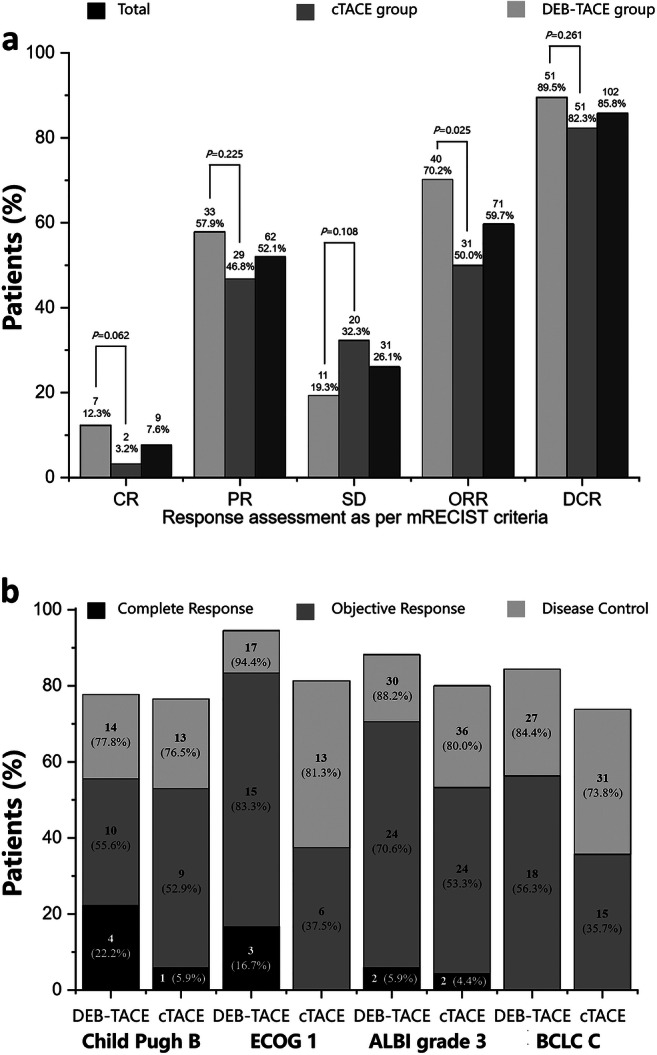
The response assessment of (**a**) the DEB-TACE and cTACE groups and (**b**) patients with more advanced diseases analyzed according to the mRECIST criteria. CR, complete response; cTACE, conventional transarterial chemoembolization; DCR, disease control rate, DCR = CR + PR + SD; DEB-TACE, drug-eluting beads transarterial chemoembolization; mRECIST, modified Response Evaluation Criteria in Solid Tumors; ORR, objective response rate, ORR = CR + PR; PR, partial response; SD, stable disease

According to the mRECIST criteria, complete response (CR), partial response (PR), stable disease (SD), PD, ORR (the sum of CR and PR rates), and DCR (sum of CR, PR, and SD rates) between the two groups are shown in Fig. 3a. In the DEB-TACE group, 7 patients (12.3%) exhibited CR, 33 (57.9%) PR, 11 (19.3%) SD, and 6 (10.5%) PD. In the cTACE group, 2 patients (3.2%) exhibited CR, 29 (46.8%) PR, 20 (32.3%) SD, and 11 (17.7%) PD. Compared with the cTACE group, the DEB-TACE group had similar DCR (89.5% vs 82.3%, *p* = 0.261), but better ORR (70.2% vs 50.0%, *p* = 0.025). Further analyses indicated that, in 92 patients with more advanced diseases (Child-Pugh B, ECOG 1, ALBI grade 3, Barcelona Clinic Liver Cancer [BCLC] C stage), the incidence of objective response was statistically higher (69.1% vs 50.8%, *p* = 0.025) in the DEB-TACE group than in the cTACE group. The greatest advantage of DEB-TACE over cTACE in terms of ORR was observed in the ECOG 1 and BCLC C stage subgroups (83.3% vs 37.5% and 56.3% vs 35.7%, respectively; Fig. 3b).

### Survival outcomes

**Fig. 4:**
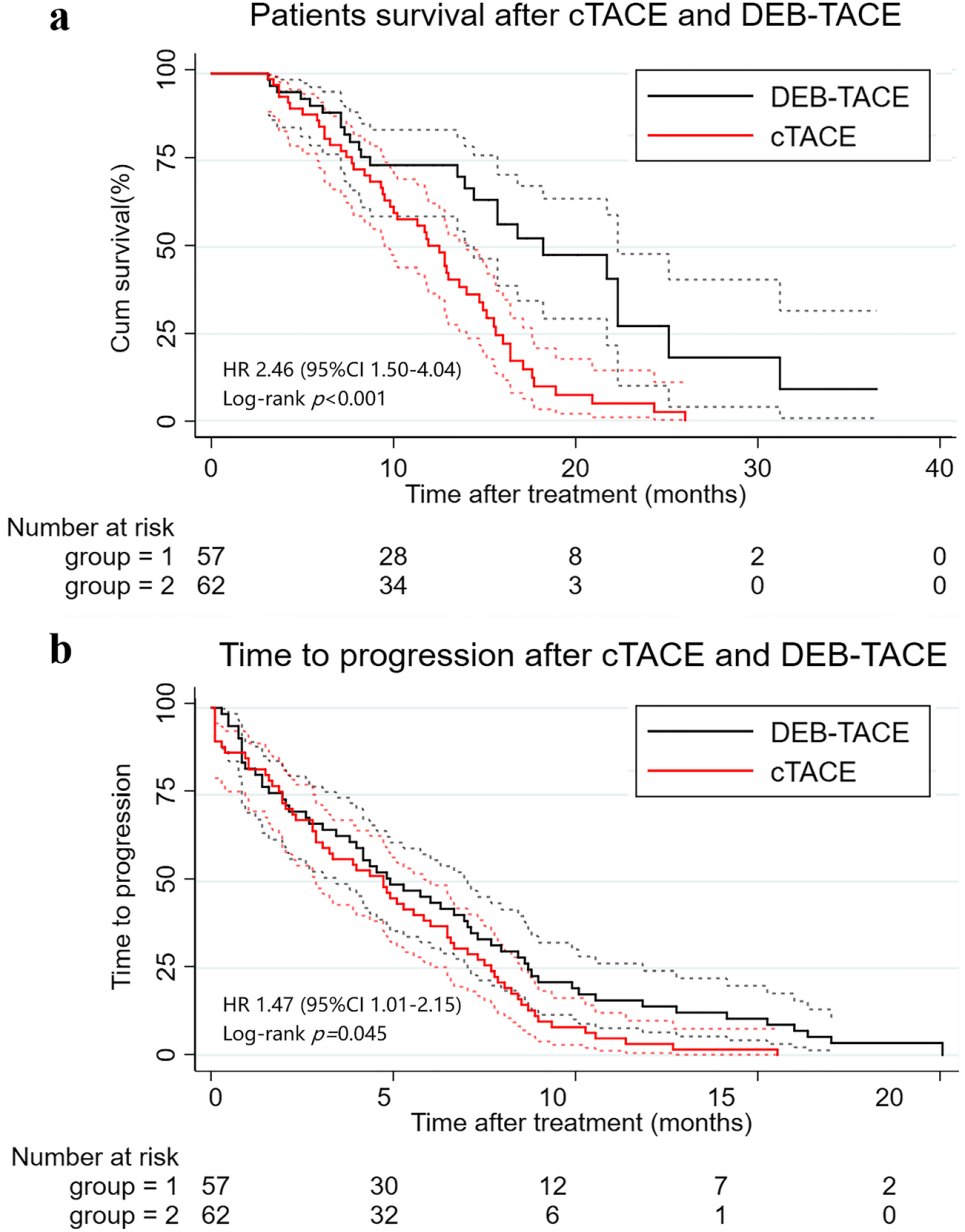
Kaplan-Meier curves show (**a**) overall survival and (**b**) time to progression of the DEB-TACE and cTACE groups. Dotted lines represent the 95% confidence interval. cTACE, conventional transarterial chemoembolization; DEB-TACE, drug-eluting beads transarterial chemoembolization; HR, hazard ratio

Of the 119 patients enrolled, 75 died of hepatic failure (61 patients), esophageal or gastric variceal bleeding (9 patients), and rupture of HCC (5 patients). The median OS of the patients in DEB-TACE group (11.4 months, 95% confidence interval [CI]: 10.1−14.0) was better than that in cTACE groups (9.1 months, 95% CI: 9.6−12.3) (hazard ratio [HR] = 2.46, 95% CI: 1.50−4.04, *p* < 0.001) (Fig. 4a). The DEB-TACE group (6.9 months, 95% CI: 5.3−8.4) was also superior to the cTACE group (5.2 months, 95% CI: 4.3−6.2) in terms of TTP (HR = 1.47, 95% CI: 1.01−2.15, *p* = 0.045) (Fig. 4b). Treatment outcomes of each institution are shown in Supplemental Table S1.

### Univariable and multivariable analyses

On univariable analysis, high AFP level (*p* < 0.001), the presence of PVTT (*p* < 0.001) and extrahepatic metastasis (*p* < 0.001), advanced BCLC stage (*p* < 0.001), and treatment of cTACE (*p* < 0.001) were significant prognostic risk factors of poor OS. Moreover, high AFP level (*p* < 0.001), the presence of PVTT (*p* < 0.001) and extrahepatic metastasis (*p* < 0.001), advanced BCLC stage (*p* < 0.001), and treatment of cTACE (*p* = 0.045) were significant determiners of poor TTP. On multivariable analysis, high AFP level (*p* < 0.001), advanced BCLC stage (*p* = 0.026), and treatment of cTACE (*p* = 0.001) were independent risk factors of poor OS, with HRs of 2.83, 4.22, and 2.38, respectively (Table 4). Moreover, only high AFP level (HR = 1.97, *p* = 0.001) remained an independent predictor of poor TTP (Table 4).

**Table 4:** Univariable and multivariable analyses of predictors of overall survival after treatment

		Overall survival				Time to progression survival		
	Univariable	Multivariable			Univariable	Multivariable		
Factor	*p* value	HR	95% CI	*p* value	*p* value	HR	95% CI	*p* value
Sex	0.118				0.670			
Age	0.655				0.841			
HBV	0.795				0.713			
ECOG score	0.364				0.999			
AFP ^*a*^	< 0.001	2.83	1.69–4.72	< 0.001	< 0.001	1.97	1.34–2.91	0.001
Child-Pugh class	0.192				0.081			
Intrahepatic tumors number	0.612				0.711			
Tumor size ^*b*^	0.077				0.347			
PVTT	< 0.001	0.64	0.19–2.12	0.468	< 0.001	1.02	0.42–2.46	0.970
Extrahepatic metastasis	< 0.001	1.39	0.74–2.62	0.304	< 0.001	1.44	0.87–2.39	0.160
BCLC stage	< 0.001	4.22	1.19–15.03	0.026	< 0.001	2.47	0.97–6.31	0.059
Treatment allocation ^*c*^	< 0.001	2.38	1.42–3.99	0.001	0.045	1.45	0.98–2.14	0.063

^*a*^AFP < 200 ng/ml or ≥ 200 ng/mL

^*b*^Tumor size, size of the largest tumor

^*c*^Treatment allocation, conventional transarterial chemoembolization or drug-eluting beads transarterial chemoembolization

*AFP*, alpha-fetoprotein; *BCLC*, Barcelona Clinic Liver Cancer; *CI*, confidence interval; *ECOG*, Eastern Cooperative Oncology Group; *HBV*, hepatitis B virus; *HR*, hazard ratio; *PVTT*, portal vein tumor thrombus

## Discussion

A patent TIPS decompresses the portal venous flow into the systemic circulation, subsequently altering hepatic portal venous perfusion. Consequently, for HCC patients who underwent a TIPS procedure, TACE is regarded as a comparative contraindication [26]. Although TACE may be suitable in a subset of patients [13, 15], this procedure may be associated with increased liver toxicity compared to similar patients without TIPS [14]. Therefore, TACE might be suggested for patients at higher risk of liver decompensation, and should liver failure occur, they may be candidates for liver transplantation [14]. However, our study showed that DEB-TACE is safe and effective in HCC patients with TIPS. DEB-TACE was found to be superior to cTACE in terms of complications, liver toxicities, ORR, TTP, and OS. Furthermore, the ORR of DEB-TACE (70.2%) was also better than that of cTACE in previous studies by Padia et al (50%) and Kuo et al (50%) [27, 28]. These local effects and survival advantages may be due to the enhanced efficacy of DEB-TACE, which sustained the drug delivery for 2−4 weeks at local tissue concentrations above the cytotoxic threshold required to kill the tumor cells.

Additionally, repeated cTACE is considered harmful to liver function. According to the PRECISION V randomized trial, postprocedural increases in liver enzyme levels, treatment-related AEs, and toxicity were remarkably lower in patients undergoing DEB-TACE than in those undergoing cTACE [11, 20]. In the post hoc analyses of patients with more advanced diseases, DEB-TACE showed significant advantages, wherein the DCR and local response improved, and better tolerability was achieved. For patients with advanced HCC, whose liver function is more likely to fail after cTACE, this finding is of particular importance, because treatment with cTACE in these patients remains controversial [14, 29]. Contrary to cTACE, the response rate of DEB-TACE in such subgroup was preserved. The causes of these advantages might include (*a*) the ability of DEB-TACE to actively keep doxorubicin hydrochloride apart from the solution and release it at a controlled and sustained rate, and (*b*) the use of particles allowing a deeper distal embolization of tumor feeding arteries, which helped to preserve the blood flow of normal liver tissues [30]. This indicates that the ameliorated tolerability of DEB-TACE allows for repeated therapeutic procedures to be performed even in patients with limited liver function, further proving the rationale of DEB-TACE, which is to effectively control intrahepatic tumors.

Our study is a multicenter study that assessed the efficacy and safety of DEB-TACE, in comparison with those of cTACE, in HCC patients after TIPS, which has not been well established in the literature. Compared with the CR rates and ORR (12.2−26% and 52.0−80.7%, respectively) reported in previous studies of HCC patients without TIPS treated by DEB-TACE [11, 20, 31], the CR rate and ORR (12.3% and 70.2%, respectively) observed in our study are comparable. However, the median OS and TTP of the DEB-TACE group in our study are 11.4 and 6.9 months, respectively, which are shorter than the outcomes of previous studies (OS = 12.3−26.1 months, TTP = 11.5−25.3 months) [32, 33]. This confirms that adequate liver function is still one of the most substantial factors in determining the survival of HCC patients. Although TIPS relieved portal hypertension, patients are still in the decompensatory period of cirrhosis, and approximately 30% of the hepatofugal flow through a TIPS was considered to adversely affect liver function [18]. Tumor number, tumor size, PVTT, and hepatic function were found to be predictors of the TACE response, which is consistent with the findings of previous studies [32, 33].

There were several limitations to our study. First, our study was retrospective. Therefore, further prospective randomized controlled investigations are needed. Second, we excluded patients with PVTT in the main trunks, who were predisposed to acute post-TACE liver decompensation because the blood flow of the main portal vein was blocked. Third, procedures of TIPS and TACE are slightly different across our five sampled institutions; thus, some effects of bias might also have an influence on our results. Last, Sorafenib is the first-line treatment of advanced HCC. However, our research did not evaluate the effects of the combination of sorafenib and TACE in post-TIPS HCC patients. A multicenter retrospective study has been carried out in our institutions aiming to identify the candidates for this combination therapy.

In conclusion, DEB-TACE is safe and effective for HCC patients with a TIPS, improves OS, ORR, and liver toxicities and is non-inferior to cTACE in terms of TTP. DEB-TACE might be a potential new therapeutic option for HCC patients with TIPS.

## Supplementary Information

ESM 1 (DOCX 1659 kb)

## Abbreviations

AEs: Adverse events
cTACE: Conventional TACE
DCR: Disease control rate
DEBDOX: Drug-eluting beads loaded with doxorubicin
DEB-TACE: Drug-eluting beads TACE
OS: Overall survival
PVTT: Portal vein tumor thrombus
TIPS: Transjugular intrahepatic portosystemic shunt
TTP: Time to progression

## Declarations

### Guarantor

The scientific guarantor of this publication is Jiaping Li (Department of Interventional Oncology, The First Affiliated Hospital of Sun Yat-Sen University, Guangzhou, People’s Republic of China).

### Methodology

• Retrospective

• Case-control study

• Performed at five multiple centers
