# Supplementary material for: Drug-eluting beads TACE is safe and non-inferior to conventional TACE in HCC patients with TIPS
Source: Eur Radiol. 2021 Apr 24;31(11):8291–301. doi: 10.1007/s00330-021-07834-9 (PMC8523393; doi:10.1007/s00330-021-07834-9)

**Supplemental Figure 1:** Computed tomography (CT) and digital subtraction angiography (DSA) images of a 50-year-old man with hepatocellular carcinoma (HCC) who underwent one session of drug-eluting beads transarterial chemoembolization (DEB-TACE) after transjugular intrahepatic portosystemic shunt (TIPS).

The red arrows indicate the main tumor in CT. The red arrowheads indicate the main tumor in DSA. The green arrowheads indicate the stents of TIPS on DSA. (A, B) Two cross-sectional and coronal-sectional contrast-enhanced CT scans for the tumor location, illustrating that the main tumor ranged 3 cm in the left lobe of the liver. (C, D) Two DSA images showing the blood flow of the portal vein before and after stent placement. (E, F) Two DSA images showing the left arterial blood supply of the main tumor in the left lobe of the liver before and after embolization. (G, H) Two cross-sectional contrast-enhanced CT scans performed at 1 and 2 months after the session of DEB-TACE, illustrating the necrosis of the main tumor and significant shrinkage of the lesion, respectively.


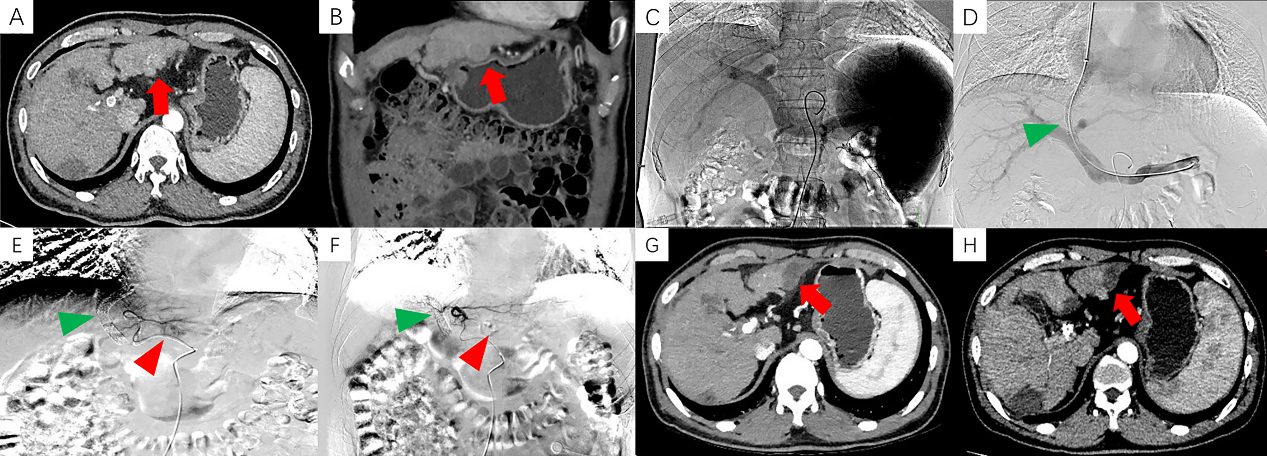


**Supplemental Figure 2:** Computed tomography (CT) and digital subtraction angiography (DSA) images of a 52-year-old man with hepatocellular carcinoma (HCC) who underwent four session of drug-eluting beads transarterial chemoembolization (DEB-TACE) after transjugular intrahepatic portosystemic shunt (TIPS).

The red arrows indicate the main tumor in CT. The red arrowheads indicate the main tumor in DSA. The green arrowheadsindicate the stents of TIPS on DSA. (A, B) Two cross-sectional and coronal-sectional contrast-enhanced CT scans for the tumor location, illustrating that the main tumor ranged 9 cm in the right lobe of liver. (C, D) Two DSA images showing the blood flow of the portal vein before and after stent placement. (E, F) Two DSA images showing the right arterial blood supply of the main tumor in the right lobe of the liver before and after embolization. (G, H) Two cross-sectional contrast-enhanced CT scans performed at 1 and 2 months after the fourth session of DEB-TACE, illustrating the necrosis of the main tumor and significant shrinkage of the lesion, respectively.


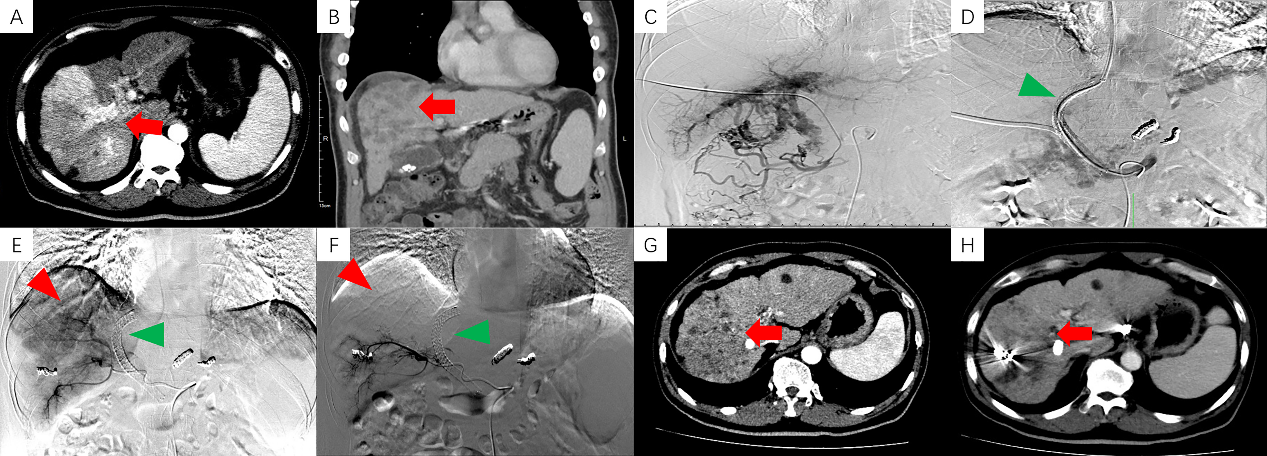

Supplement: Supplementary file 1 — (DOCX 1659 kb) [file 330_2021_7834_MOESM1_ESM.docx]
